# Performance of All-Solid-State MoO_x_ pH Sensors Prepared via Thermal Oxidation for Robust Applications

**DOI:** 10.3390/s25030611

**Published:** 2025-01-21

**Authors:** Djouhar Aoubida, Stephanie Betelu, Johan Bertrand, Quoc Nghi Pham, Diana Dragoe, Nita Dragoe, Ioannis Ignatiadis

**Affiliations:** 1 BRGM (French Geological Survey), 45060 Orleans, France; s.betelu@brgm.fr; 2 ICMMO (Institute of Molecular Chemistry and Materials), 91400 Orsay, France; quoc-nghi.pham@universite-paris-saclay.fr (Q.N.P.); diana.dragoe@universite-paris-saclay.fr (D.D.); nita.dragoe@universite-paris-saclay.fr (N.D.); 3 ANDRA (French National Radioactive Waste Management Agency), 92298 Châtenay-Malabry, France; johan.bertrand@andra.fr

**Keywords:** all-solid-state electrodes, pH sensor, redox potential, nuclear waste, geological disposal monitoring, thermal oxidation, molybdenum oxide electrode

## Abstract

In this study, we investigated the morphology, chemical composition and pH measurement performance of MoO_x_ electrodes prepared via thermal oxidation and submitted to hydration in milliQ water. Surface analysis using SEM-EDS and XPS revealed that the hydrated MoO_x_ film is composed of different oxidation states of Mo (Mo (IV), Mo (V) and Mo (VI)), influencing its electrochemical behavior. A hydration period of 45 days was required for the electrode to achieve a response approaching the Nernstian model (−58 mV/pH), while extended hydration of up to 70 days enhanced its stability and sensitivity. The electrode’s performance was assessed under various conditions, including in the presence and absence of oxygen and in anaerobic conditions with the presence of sulfides. Oxygen absence increased sensitivity and lowered the experimental standard potential (E^0^_Exp_) due to the effect of oxygen vacancies. Low sulfide concentrations had minimal impact on electrode performance, although higher concentrations may slightly decrease the electron transfer efficiency due to the complex formation. The pH sensing mechanisms of MoO_x_ electrodes are also discussed.

## 1. Introduction

In Europe, deep geological disposal is the preferred option for nuclear waste management to isolate radioactive waste from human environments. In France, the planned deep geological disposal facility for high-level and intermediate-level long-lived radioactive waste, known as “Cigeo”, will be constructed 500 m underground in the Callovo–Oxfordian geological formation (Cox). Overseen by the French National Radioactive Waste Management Agency (Andra), the Cox formation is a 130-m-thick stratigraphic unit primarily composed of clay, with origins tracing back approximately 160 million years. Located at depths between 400 and 600 m, this formation is a hydrogeological environment characterized by high water saturation and extremely low permeability, porosity and hydraulic conductivity.

The temperature, pH and CO_2_ partial pressure of the Cox pore-water solution are constant at 25 °C, 7.3 (±0.1) and 8.10^−3^ atm, respectively [1]. Anoxic conditions prevail in the Cox formation. Within the mineralogical assemblage [2,3], geochemical models predict E_H_ values ranging from −180 to −200 mV, corresponding to an equilibrium between pyrite and pore-water sulfate [S^(+VI)^] concentrations, and iron-bearing phases such as Fe-bearing carbonates or nanogoethite [3,4,5,6].

Andra is in charge of the long-term radioactive waste management in France. The radioactive wastes will be placed in horizontal pipes made of Carbon Steel API-5L-X65 (CSX65), separated from the Cox by a cemento-bentonitic filling material (CBFM). The technical specifications for developing monitoring techniques are based on the following: (i) requirements arising from the specific nature of the parameters that need to be measured in key thermal-hydraulic-mechanical-chemical and radiological (THMCR) processes; and (ii) requirements related to the minimum accuracy and long-term stability of the monitoring methods—considering that there will be little or no access for re-calibrating the sensors—for accurately monitoring the evolution of the near-field around the radioactive waste, including Cox pore water alone and in contact with carbon steel CSX65 and an alkaline CBFM (pH > 11.7).

Some constraints specific to on-site conditions must be considered for developing the sensors [7]:

1. The wide range of redox potentials over the Pourbaix diagram due to the following: (i) gas emissions such as O_2_ from excavation, H_2_ from the release of radioactive waste and metal corrosion, CO_2_ from organic-matter degradation, H_2_S from the activity of sulfate-reducing bacteria (SRB), or CH_4_ from the activity of methanogenic bacteria; (ii) sulfide (HS^−^/S^2−^) production due to the activity of SRB.
2. The temperature will increase due to radioactive disintegration (25 °C ≤ T ≤ 90 °C).
3. The initial pH of the Cox pore water will decrease due to pyrite oxidation. Then, a progressive alkalization of the Cox pore water will occur due to its contact and interaction with CBFM (pH greater than 11.7). Thus, the pH domain to monitor ranges from 4.0 to 13.0.

The two key parameters for monitoring the above parameters are thus pH and redox potential [8] as they effectively reflect physical, chemical and electrochemical (redox) transformations. To ensure the safety and reversibility of the nuclear waste storage, reliable sensors are essential for monitoring changes in the near-field environment [7,9,10]. The objective is to design, create and optimize a robust multi-parameter probe for the on-site monitoring of pH (±0.5 pH unit) and redox potential (±50 mV) in order to ensure the long-term safety of the operation [7]. The multiplication of electrodes is essential for consistently and reliably monitoring the evolution of the aqueous environment over time. Evaluating the durability and performance of these sensors requires testing under conditions that replicate real-world scenarios, such as using a glove box to simulate low oxygen controlled atmosphere and, additionally, to investigate the impact of sulfides, which may arise from bacterial activity in confined environments [7].

The glass electrode is the most widely used pH sensor. However, it has several limitations, including fragility, lack of long-term stability, high impedance and difficulties in miniaturization for specific applications. As a result, new types of electrodes have been developed to respond to the requirements of measuring pH in different types of solutions, such as corrosive solutions, alkaline solutions and HF solutions. These innovations include ion-sensitive field-effect transistor (ISFET) pH sensors [11,12], fiber optic pH sensors [13,14,15], hydrogel membrane pH sensors [16,17] and metal oxide pH sensors [8,18,19].

Metal oxide sensors have attracted particular attention because of their advantages, such as fast response times and high stability. The choice of metals is mainly based on their electrochemical behavior and stability across the pH range (4–13). Several research groups have investigated new metal oxides for use as pH electrodes, including Ir/IrO_x_ [20,21,22,23], Pt/PtO_2_ [24,25,26], Ru/RuO_2_ [27] Ti/TiO_2_ [28], Sb/Sb_2_O_3_ [7], Pb/PbO_2_ [29] and CeO_2_ [9].

Beyond traditional metal oxide electrodes, electrodes fully composed of metal oxides on non-metallic substrates are also used. The pH response of these electrodes is often attributed to the presence of the mixed oxide-hydroxides within the film [30,31,32]. For some materials, further research is needed to develop coatings that can minimize mechanical stresses on the sensitive layer during measurements in basic environments. Electrodes such as Sb/Sb_2_O_3_ [7] have been tested under controlled conditions that replicate environments likely to be encountered in storage facilities [10].

On the other hand, molybdenum oxides also have considerable potential due to their different oxidation states. Molybdenum exhibits oxidation states from (+II) to (+VI), enabling it to form a wide range of oxide compounds [33], a capability linked to its delocalized 4d electrons. The most important and common are molybdenum trioxides (MoO_3_) and dioxides (MoO_2_). The nature and composition of surface oxides vary according to the nature of the medium (air, aqueous, non-aqueous), the pH of the electrolyte, the potential of the electrode and the operating parameters, such as temperature and pressure [33]. MoO_3_ has a perovskite-type structure, with oxygen vacancies. The structure of molybdenum allows the efficient insertion or intercalation of ions like H^+^ within this structure. This intercalation process modifies the optical properties of the material and its charge storage capacity. Molybdenum is therefore very useful in applications such as for batteries and electrochromic devices [34].

However, the use of molybdenum is still relatively limited for pH applications compared with these more commonly used oxides (RuO_x_ and IrO_x_). For example, Shuk et al. [35] developed pH sensors based on molybdenum bronzes, such as Na_0.9_Mo_6_O_17_ and Li_0.9_Mo_6_O_17_. The Na-molybdenum-oxide bronzes pH sensors exhibited near ideal Nernstian behavior in the pH range of 3 to 9 [35]. Another example is the molybdenum diselenide/nitrogen doped graphene oxide screen-printed electrode (MoSe_2_/NGO), developed by Poorahong et al., which demonstrated high stability and reproducibility over a wide pH range from 2 to 14 [36].

In this study, MoO_x_ electrodes were prepared via thermal oxidation and then hydrated in milliQ water. The surface morphology and chemical composition of the MoO_x_ films were studied using SEM-EDS and XPS. In addition, the influence of hydration on the evolution of the E–pH relationship of the electrodes was analyzed and compared. Electrochemical performance of the electrodes, as pH sensors, was evaluated under calibration conditions simulating field expectations. Their reliability and robustness were assessed through electrochemical measurements at 25 °C, under atmospheric pressure and/or in a glove box (GB).

## 2. Materials and Methods

### 2.1. Sample Preparation

**Figure 1:**
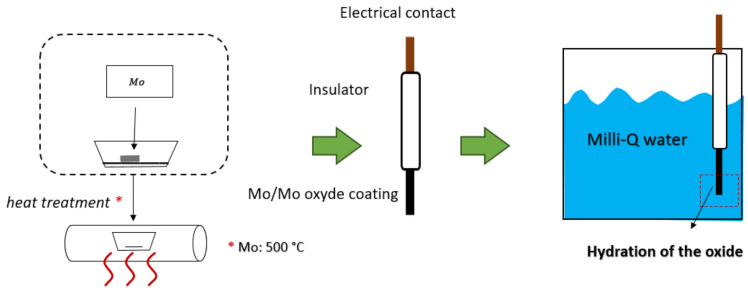
Preparation of the MoO_x_ electrode using thermal oxidation and hydration.

Molybdenum wires (diameter 1.5 mm, purity 99.9%, Merck, Rahway, NJ, USA) were used. The wires were polished with a series of abrasive papers (1000#, 1500# and 2000#) for around 2 min to remove the surface oxide layer. Next, the wires were ultrasonically cleaned in milliQ water (resistivity = 18.2 MΩ·cm) and ethanol, respectively. Then, the cleaned molybdenum wires were oxidized at 500 °C for one hour. After cooling to room temperature, the wires were rinsed again with milliQ water. For electrode fabrication, one end of the oxidized molybdenum wire was affixed to a copper conductor via soldering. The connection was subsequently insulated using heat-shrink tubing, ensuring that precisely 3 mm of the oxidized molybdenum surface remained exposed for electrochemical interaction. The electrodes were then immersed in milliQ water at 25 °C for hydration, as shown in Figure 1.

### 2.2. Supporting Electrolytes: Buffers and Solutions

Experiments were conducted at a constant temperature of 25.0 ± 0.1 °C, either at atmospheric pressure or within a thermo-regulated glove box (pN_2_ = 1 atm, PO_2_ ≈ 10^−6^ atm) [7]. The electrodes’ responses to pH variations were extensively examined by immersing them in various buffered solutions prepared using deionized water with a resistivity of 18 MΩ·cm. Each buffered solution had an ionic strength of 0.05 M, achieved by adding a precise amount of NaCl to closely match the ionic strength and salinity of the in-situ Cox pore water [6]. For pH > 10.8 (the upper limit of carbonate-based buffers), the pH of the solutions was adjusted by adding 1 M sodium hydroxide. Specific buffered solutions used, along with their corresponding effective pH ranges, are outlined in Table 1. In all experiments investigating the influence of pH on the open circuit potential of the electrodes, measurements were conducted within a pH range between 4 and 13.

In addition to the calibration curves constructed in the absence of sulfides, we performed calibration curves in the presence of sulfides at various concentrations (ranging from 10^−7^ to 10^−3^ M). Sulfides exist in various forms: H_2_S_(g)_, H_2_S_(aq)_ and HS^−^_(aq)_, S^2−^_(aq)_. The speciation of sulfides in solution depends on the pH value. Since the pH of the Cox pore water is close to neutral (7.0 < pH < 7.4 at 25 °C), sulfides will predominantly be in the form of H_2_S_(g)_, H_2_S_(aq)_ and HS^−^_(aq)_. It is crucial to consider the expected pH variations during the operational phase of the storage facility, as they will impact sulfide speciation. This is why we decided to investigate the electrode behavior in the presence of sulfides at various concentrations and pH values in the laboratory.

### 2.3. Characterization of MoO_x_/Mo Film

#### 2.3.1. Scanning Electron Microscopy (SEM)

The morphology of MoO_x_/Mo coatings was observed with a TESCAN (Tescan Group, a.s., Brno, Kohoutovice, Czech Republic) scanning electronic microscope (SEM) with an operating energy of 15 kV, coupled with energy dispersive X-ray spectroscopy (EDS) to characterize the elemental composition.

#### 2.3.2. X-Ray Photoelectron Spectroscopy (XPS)

The XPS spectra were recorded by a Thermo Fisher Scientific (Courtaboeuf, Les Ulis, France) spectrometer equipped with an Al K alpha monochromatic high-energy radiation source (hʋ = 1486.7 eV) and a hemispherical analyzer operating in Constant Analyser Energy (CAE) mode. Additionally, the X-ray spot size measured 200 µm, resulting in an irradiated area of approximately 0.5 mm^2^. Binding energies were calibrated based on the C 1s peak (284.88 eV). XPS data were analyzed using CASA XPS software version 2.3.25PR1.0 (Clearwater, FL, USA). A Gaussian-Lorentzian GL (30) peak shape was employed to deconvolve the C1s and O1s peaks, while a Modified Lorentzian Peak Function LF (0.8, 1.35, 280) was used for the Mo 3d peaks. Survey scans were conducted with a pass energy of 200 eV and a step size of 1 eV. High-resolution windows were acquired with a pass energy of 50 eV and a step size of 0.1 eV.

### 2.4. Potentiometric and pH Measurements

The study of potential variations of Mo-MoO_x_ based electrodes was conducted in double-walled Pyrex glass eletrochemical cells. These cells were connected to a thermostatic bath to maintain a constant temperature of 25 °C. A data acquisition unit (Keithley Instruments, model 2700, Cleveland, OH, USA), handled by a computer via KickStart version 2.7.0 software, was used to record potential variations every 15 s for 10 to 15 min. Open-circuit potential measurements of the electrodes were conducted relative to Ag/AgCl with 3 M KCl (Radiometer Analytical REF201, Hach Lange GmbH, Düsseldorf, Germany) reference electrode and were subsequently converted and referenced versus the standard hydrogen electrode (SHE). Additionally, a pH meter (OrigaMeter OpH218 from Origalys, Rillieux le Pape, France), coupled with a commercial pH electrode (OGPH203, OrigaSens, from Origalys), which was also connected to the data acquisition unit, was employed to confirm measured pH values. Data translation was facilitated by a computer.

As otherwise stated, all the experiments were made in triplicate. Each experiment had its own measurements. Regardless of the experiment, pH reached a stable value in a few seconds. OCP reached a stable potential in the range of 1 to 2 min. For each experiment, the last ten values were used to calculate the mean and the relative standard deviation of OCP and pH. For each investigated pH value, the mean and standard deviation were calculated for ten measurements repeated three times (x = 10; N = 3). We have chosen to present only the first among the three experiments in the corresponding figure and we added the statistical treatments in a separate table.

### 2.5. Electrochemical Impedance Spectroscopy (EIS)

The impedance of the all-solid-state MoO_x_/Mo electrode was measured at room temperature using a Model 2273 potentiostat–galvanostat (AMETEK, Inc., Berwyn, PA, USA), interfaced with a PC system and controlled by PAR’s PowerSuite v.2.58 software, in buffer solutions with pH values of 3.98, 6.98 and 10.8 using a three-electrode configuration. The MoO_x_/Mo electrode served as the working electrode, a platinum grid served as the counter electrode, and an Ag/AgCl KCl 3M electrode (Radiometer Analytical REF201, Hach Lange GmbH, Düsseldorf, Germany) served as the reference electrode. An AC voltage of 10 mV (rms) was applied, and the frequency range varied from 1 MHz to 0.1 mHz. The data were fitted using Zsimpwin 3.60 software (Echem software, Ann Arbor, MI, USA).

## 3. Results and Discussion

### 3.1. Characterization of MoO_x_/Mo Fil

#### 3.1.1. SEM Analysis

**Figure 2:**
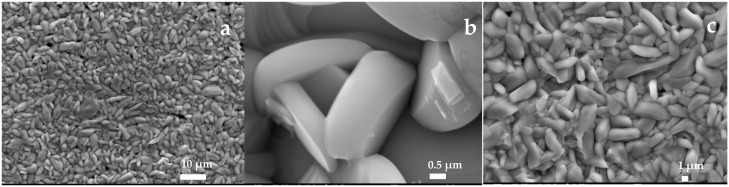
Representative SEM images of MoO_x_ films before hydration prepared using thermal oxidation at 500 °C. (**a**) magnification: ×1300, scale given by the white bar: 10 µm, (**b**) magnification: ×15, scale given by the white bar: 0.50 µm, (**c**) magnification: ×3, scale given by the white bar: 1 µm.

As shown in Figure 2, after the oxidation of the molybdenum wire at 500 °C for 1 h, SEM images indicate the formation of molybdenum oxides on the surface. At low magnification, a dense layer of small particles, probably MoO_3_ crystals, is observed. The high-oxygen atmosphere at 500 °C favored the complete oxidation of MoO_2_ to MoO_3_, which is in agreement with previous observations on the rapid transformation of MoO_2_ to MoO_3_ under such conditions [37].

With increasing magnification, more defined structures appear, suggesting progressive crystallization. The particles adopt irregular shapes on a microscopic scale, characteristic of molybdenum oxides formed during thermal oxidation [38]. The surfaces become smoother and more angular as crystal growth progresses.

**Figure 3:**
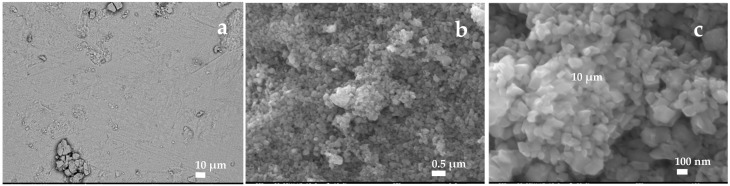
Representative SEM images of MoO_x_ films after hydration prepared via thermal oxidation at 500 °C. (**a**) magnification: ×450, scale given by the white bar: 10 µm, (**b**) magnification: ×15, scale given by the white bar: 0.50 µm, (**c**) magnification: ×50, scale given by the white bar: 100 nm.

However, after hydration in Milli-Q water for 70 days, at the scale of 10 µm (comparison between Figure 2a and Figure 3a), it is difficult to observe significant morphological differences before and after hydration, as the resolution is not sufficient. The comparison between Figure 2b and Figure 3b shows that the structure changes significantly after hydration: particles become smaller, more irregular and the surface becomes more porous and granular. Hydration modifies the morphology of the material, requiring finer resolutions to visualize these changes (see the comparison between Figure 2b and Figure 3b,c).

These observations could indicate that the oxides initially formed have partially dissolved in the water and then recrystallized as hydrated oxides, resulting in a loss of crystallinity [39] and an increase in porosity.

**Figure 4:**
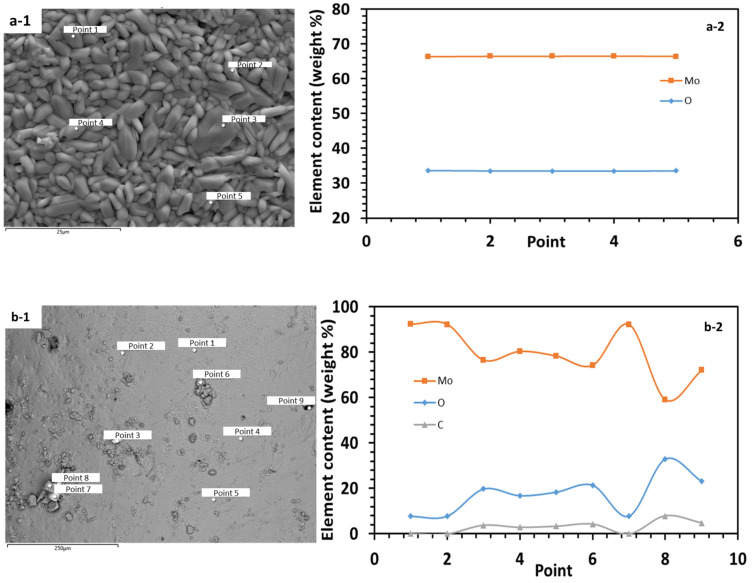
Elemental Distribution Analysis (**a-2**,**b-2**) at locations marked as points distributed over the surface of SEM images (**a-1**,**b-1**) of the MoO_x_ film before and after hydration, produced via thermal oxidation.

Additionally, the line scan of Energy Dispersive Spectrometry (EDS) is performed to analyze the chemical composition of the MoO_x_ film. In Figure 4, the EDS analysis points are shown as evenly distributed markers on the surface.

In Figure 4(a-1), points 1–5 show that the sample is predominantly composed of molybdenum with significant oxygen content, indicating the formation of oxides. This suggests that the molybdenum has effectively oxidized to form a stable oxide layer, likely MoO_3_. The composition appears homogeneous, reflecting a consistent distribution of oxygen within the material structure.

In contrast, Figure 4(b-1) shows that points 3, 4, 5, 6, 8 and 9 have relatively high oxygen and low molybdenum contents. However, at points 1, 2 and 7, the oxygen weight content is approximately 8%, indicating that the surface is largely covered with MoO_x_, with varying values of “x” at different locations due to the partial dissolution of molybdenum and the formation of hydrated phases. The appearance of carbon in several points could indicate minimal contamination by organic residues present in the air particles, as the recipient was exposed to air during the hydration process.

#### 3.1.2. XPS Analysis

**Figure 5:**
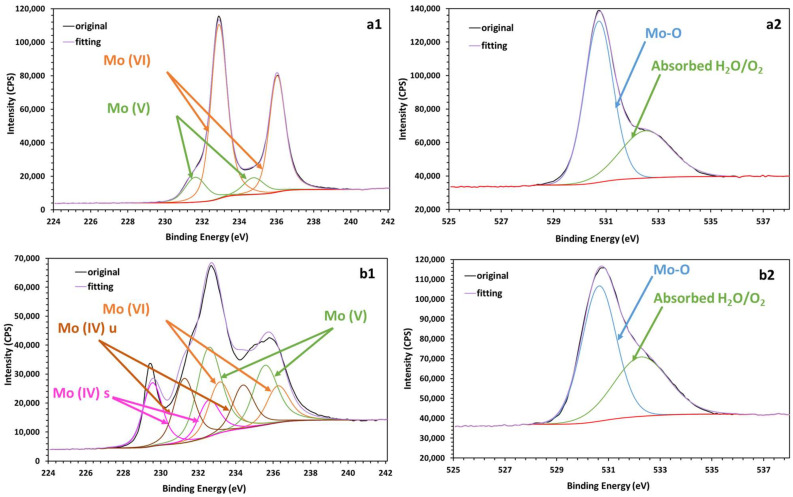
XPS of a MoO_x_ film, prepared by thermal oxidation. (**a1**,**a2**): deconvolution of Mo 3d and O 1s spectra before hydration; (**b1**,**b2**): deconvolution of Mo 3d and O 1s spectra after hydration.

The chemical composition of the MoO_x_ film, obtained via thermal oxidation, is studied using XPS before and after hydration. For deconvolution of the 3d Mo peaks, the splitting of the 3d doublets is constrained to be very close to 3 eV; the doublets have very similar maximum widths at half maximum (FWHM), with an area ratio of 3:2 (3d5/2 compared with 3d3/2). Figure 5 shows the Mo 3d5/2, Mo 3d3/2 (a1, b1) and O 1s (a2, b2) spectra of the MoO_x_ thin film.

The deconvolution of the Mo 3d peaks (Figure 5(a1)) [40,41] suggests the existence of Mo (VI) and Mo (V), with fitting details summarized in Table 2. Double peaks at binding energies of 232.88 eV and 236.01 eV correspond to the 3d (5/2) and 3d (3/2) states of Mo (VI), respectively [40,41,42,43]. Similarly, peaks at binding energies of 231.60 eV and 234.73 eV correspond to the 3d (5/2) and 3d (3/2) states of Mo (V) [40,44]. The presence of Mo (V) is attributed to a defect structure caused by the reduction of Mo (VI) under the X-ray beam [41]. Predominantly, Mo (VI) states confirm the presence of MoO_3_ on the surface. The deconvolution of the O 1s spectrum before hydration (Figure 5(a2)) indicates oxygen bound in molybdenum oxides (Mo-O) (530.73 eV) [42,43] and absorbed H_2_O/O_2_ (532.52 eV) [45].

After hydration, the Mo 3d peaks (Figure 5(b1)) shift slightly, with binding energies at 233.10 eV (3d (5/2), Mo(VI)), 236.22 eV (3d (3/2), Mo (VI)), 232.54 eV (3d (5/2), Mo (V)) and 235.54 eV (3d (3/2), Mo (V)), respectively. We also observe the appearance of Mo (IV) peaks, which form a complex Mo 3d5/2 peak with a two-component structure. The sharper, slightly asymmetric main peak at 229.53 eV and the broader peak at 231.23 eV, with higher binding energy, are attributed to screened and unscreened final states [40,42]. In addition, Mo 3d (3/2) appears at corresponding energies, with 232.53 eV for the unscreened state and 234.36 eV for the screened state [40,42], confirming the complexity of the Mo (IV) electronic structure.

The deconvolution of the O 1s spectrum (Figure 5(b2)) shows a substantial increase in the peak at 532.5 eV (H_2_O/O_2_), indicating increased adsorption of water molecules to the surface. This evolution is also combined with a slight decrease in the contribution at 530.7 eV (Mo-O), which could be explained by a partial dissolution of MoO_3_, and which is followed by the formation of hydrated phases such as MoO_3_-H_2_O.

These results indicate that hydration causes a reorganization of the surface, marked by the formation of hydrated phases due to increased water adsorption, while modifying the electronic states of molybdenum. Moreover, as MoO_x_ is an electrochemical pH sensor, the surface H_2_O absorbed should improve the sensitivity of interfacial reactions with H^+^ in solution, which controls sensor performance.

### 3.2. Response Mechanism of MoO_x_ Electrode

#### 3.2.1. SEM Analysis

**Figure 6:**
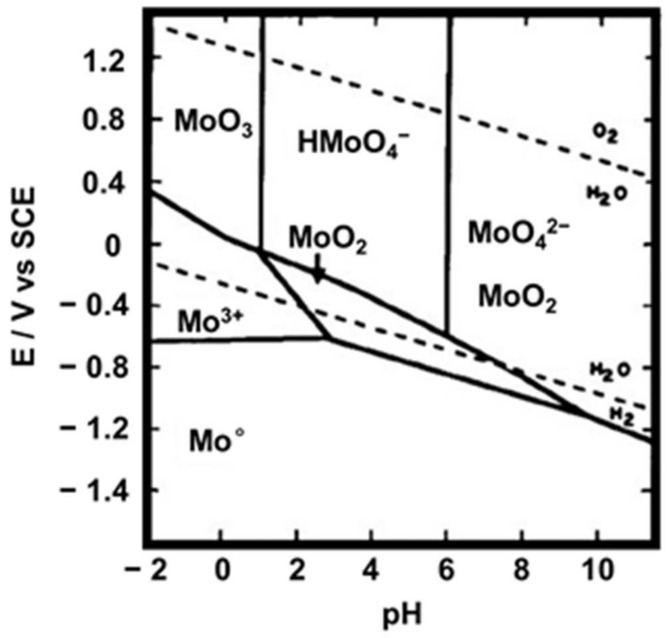
Potential–pH diagram of a Mo-H_2_O system. Dotted lines correspond to the water stability limits: oxidation of H_2_O to O_2_ and reduction of H_2_O to H_2_.

The combination of SEM-EDS and XPS provided information on the oxidation states, elemental composition and electrochemical stability of the molybdenum species. Using these characterizations and the Pourbaix diagram (Figure 6) [33], we proposed a detailed mechanism for the reactions involved in the hydrous reduction of MoO_3_.

#### 3.2.2. Oxidation of Molybdenum to Form MoO_3_

Firstly, we oxidized molybdenum in a furnace at 500 °C to form MoO_3_ according to the following reaction:

$${Mo}_{\left(s\right)}+\frac{3}{2}O_{2}\to \;{MoO}_{3\left(s\right)}$$

#### 3.2.3. XPS Analysis Before Hydration and Electron Beam Effect

Before hydration, XPS measurements revealed a partial reduction of Mo (VI) to Mo (V), probably due to interaction with the electron beam during analysis. This reduction is illustrated by the following reaction:

$${2MoO}_{3\left(s\right)}\to \;{Mo}_{2}O_{5}+\frac{1}{2}O_{2\left(g\right)}$$

#### 3.2.4. Redox Reaction Between Mo (VI) and H_2_O

A redox reaction occurs between Mo (VI) and water during hydration, leading to the formation of MoO_2_ and oxygen, as described by

$${2MoO}_{3\left(s\right)}\to \;{2MoO}_{2}+O_{2\left(g\right)}$$

The partial reduction of Mo (VI) after hydration is responsible for the creation of oxygen vacancies into a Mo_2_O_5_(s) octahedral-based structure contain a mixture Mo (VI) and Mo (IV).

#### 3.2.5. Hydration and Acid-Basic Reactions of MoO_3_ into H_2_O

The hydration and acid-base reactions of MoO_3_ in water occur according to the following reactions:

$${MoO}_{3\left(s\right)}+H_{2}O\;\to \;{HMoO}_{4}^{-}+H^{+}\;{HMoO}_{4}^{-}\to {MoO}_{4}^{2-}+H^{+}$$

The dissolution of MoO_3_ in water leads to the formation of soluble compounds such as HMoO_4_^−^ and MoO_4_^2−^. This dissolution creates micro-spaces within the solid structure, which probably increases the material’s porosity. OCP measurements conducted after 70 days of hydration (Section 3.3.1) demonstrate that this change enhances the sensor’s sensitivity. The increased porosity facilitates the interaction of the electrolyte with the material, improves ion mobility and provides better access to the reactive zones, resulting in improved electrochemical performance. However, this is not an issue, as thermodynamics predicts the formation of more stable species, though kinetics are not considered. Mo oxide is relatively stable over time, showing no troubles under the conditions studied. There is enough Mo still available to provide electrons for the Mo (VI)/Mo (V) redox reactions in the Mo_2_O_5_ structure.

#### 3.2.6. Redox Reaction Between Mo (IV) or Mo and O_2_

Finally, the oxidation-reduction reactions involving Mo (IV) or Mo and oxygen in the presence of water are illustrated below:

$${2MoO}_{2}+O_{2\left(g\right)}+2H_{2}O\to \;2{HMoO}_{4}^{-}+{2H}^{+}\;2Mo+O_{2\left(g\right)}+H_{2}O\to {MoO}_{2(s)}+{4H}^{+}$$

#### 3.2.7. Proposed Redox Mechanisms for pH Sensing

We have identified the half-reactions of the Mo (VI)/Mo (IV) redox couple, involving an exchange of protons (H^+^) and which are crucial for the development of our pH sensor. The proposed redox reactions are as follows, with an indication of the pH range in which they take place, as well as the number of electrons exchanged, which helps to explain the Nernstian and super-Nernstian response, which we will discuss in the next part of our study.

(1) $${MoO}_{3}\left(s\right)+2e^{-}+2H^{+}\;\Leftrightarrow \;{MoO}_{2(s)}+H_{2}O\;\mathrm{into}\;{{Mo}_{2}O}_{5\left(s\right)}\;\;\;0\leq \mathrm{pH}\leq 14\;({1e}^{-}\Rightarrow 1\;H^{+})\ldots$$

(2) $${HMoO}_{4}^{-}+2e^{-}+3H^{+}\;\Leftrightarrow \;{MoO}_{2(s)}+2H_{2}O\;\;\;\;\;\;\;2\leq \mathrm{pH}\leq 6\;({1e}^{-}\Rightarrow 1.5\;H^{+})\ldots$$

(3) $${MoO}_{4}^{2-}+2e^{-}+4H^{+}\;\Leftrightarrow \;{MoO}_{2(s)}+2H_{2}O\;\;\;\;\;\;\;6\leq \mathrm{pH}\leq 13\;({1e}^{-}\Rightarrow 2\;H^{+})\ldots$$

(4) $${MoO}_{4}^{2-}+6e^{-}+8H^{+}\;\Leftrightarrow \;Mo+4H_{2}O\;\;\;\;\;\;\;\;\;\mathrm{pH}\geq 10\;({1e}^{-}\Rightarrow 1.33\;H^{+})\ldots$$

### 3.3. Response of MoO_x_ Electrodes in pH Buffer Solutions

#### 3.3.1. Influence of Hydration

The hydration treatment plays a critical role in enhancing the stability of MoO_x_ electrodes fabricated using thermal oxidation. Our findings indicate that the hydration process is essential for achieving consistent performance over time.

**Figure 7:**
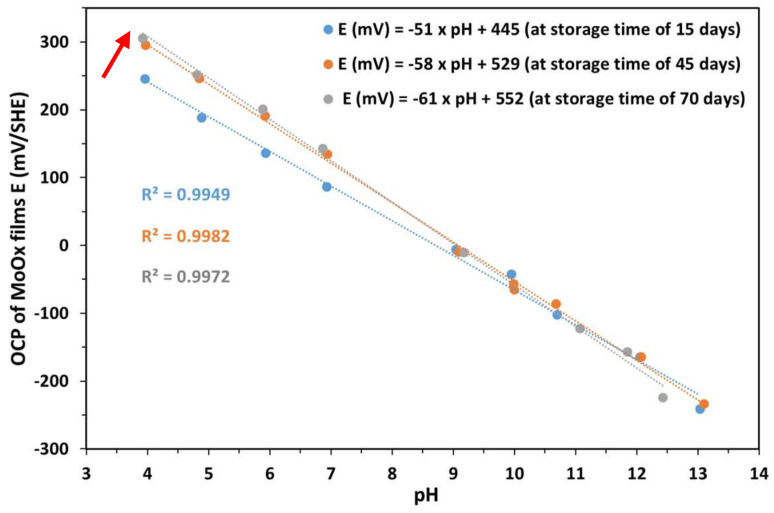
Eh-pH diagram of an MoO_x_ electrode; measurements taken at atmospheric pressure at different storage times. The arrow shows the evolution of E^0^_Exp_ as a function of hydration time.

Figure 7 shows the evolution of the open circuit potential (OCP) of MoO_x_ films as a function of pH, measured at different storage durations, in milliQ water, of 15, 45 and 70 days, under atmospheric conditions (in air). There was a progressive increase in the sensitivity of the electrode and a shift in the experimental standard potential (E^0^_Exp_) with immersion time.

- After 15 days, the slope was −51 mV/pH, slightly below the Nernstian theoretical slope of −59.2 mV/pH, with an E^0^_Exp_ of 445 mV.
- After 45 days, the slope increased to −58 mV/pH and the E^0^_Exp_ increased to 529 mV, approaching the Nernstian response.
- After 70 days, the slope increased to −61 mV/pH, slightly exceeding the theoretical Nernstian value, and the E^0^_Exp_ reached 552 mV, suggesting a super-Nernstian response.

The evolution in both E^0^_Exp_ and sensitivity results from the increase in measured potentials in the acidic pH range (between 4 and 7). This can be attributed to a process of continuous hydration of the MoO_x_ films, which probably favored the stabilization of redox states at the surface and improved the exchange of H^+^ ions. This hydration caused an increase in dissolved species, such as ${HMoO}_{4}^{-}$ and ${MoO}_{4}^{2-}$, which vary in concentration as a function of pH. In the coating, their presence is directly responsible for the increase in E^0^_Exp_.

In addition, reactions 2 and/or 3 participate in the redox process by increasing the amount of H^+^ protons involved in the redox reactions per electron exchange, which improves the slope and promotes a more favorable electrochemical response.

In alkaline pH, more specifically at pH ≥ 10, reaction 4 becomes predominant. This reaction, characterized by an exchange of 1.33 H^+^ per electron, shows the stability of the MoO_x_ electrode in basic environments. As a function of pH, the different redox half-reactions (from 1 to 4) of the Mo (VI)/Mo (IV) pair involve a variation in the number of H^+^ protons exchanged per electron, thus influencing the sensitivity of the pH sensor. Based on the experiments carried out, we decided to use a hydrated molybdenum electrode for further research, which was immersed in Milli-Q water for 70 days.

Table 3 presents the statistical treatments of the three experiments on each hydration time (15, 45 and 70 days) on the sensitivity (mV/pH) and the E^0^_Exp_ (mV). The standard deviations observed for sensitivity (≤±0.9 mV/pH) and E^0^_Exp_ (≤±9.0 mV) indicate good reproducibility of the measurements across all hydration times.

#### 3.3.2. Reversibility of the Hydrated Molybdenum Electrode

**Figure 8:**
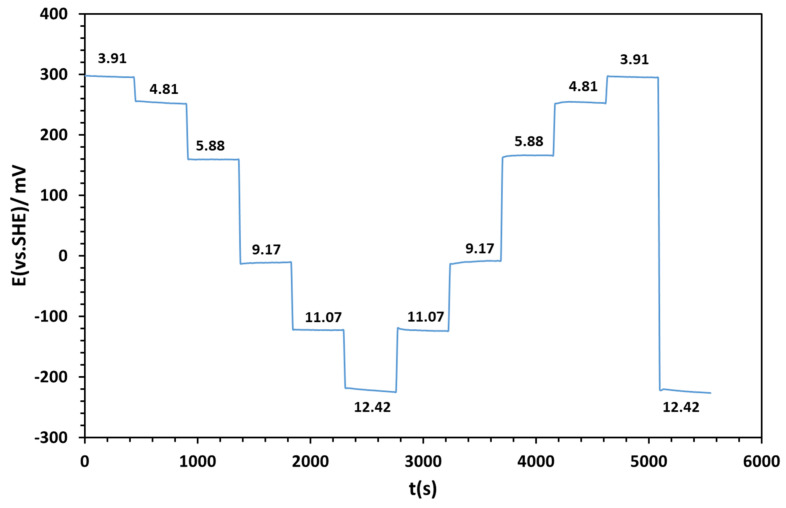
Study of OCP stability of Mo 500 °C as a function of time, with reversible pH values. Hysteresis for pH = 3.91 from rise and fall is 3 mV.

**Figure 9:**
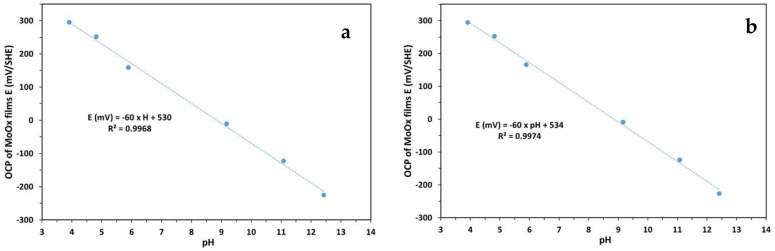
pH electrode calibration curves for MoO_x_ as pH values decrease (**a**) and increase (**b**).

In Figure 8, the molybdenum electrode after 70 days of hydration exhibits good reversibility. The E-pH relationship of the electrode synthesized via thermal oxidation is E (mV) = −60 × pH + 530 (R^2^ = 0.9968) (Figure 9a) when varying the pH from acidic to alkaline, and E (mV) = −60 × pH + 534 (R^2^ = 0.9974) (Figure 9b) when varying the pH from alkaline to acidic. In other words, the molybdenum electrode after hydration shows a rapid response when a significant change in pH from 3.93 to 12.47 is made.

Table 4 presents the average OCP values for the rise and fall phases of pH according to Figure 8.

#### 3.3.3. Performances of the Hydrated MoO_x_ Electrode in a Glove Box (GB)

We evaluated the MoO_x_ electrode in a glove box to observe its behavior under low-oxygen conditions.

**Figure 10:**
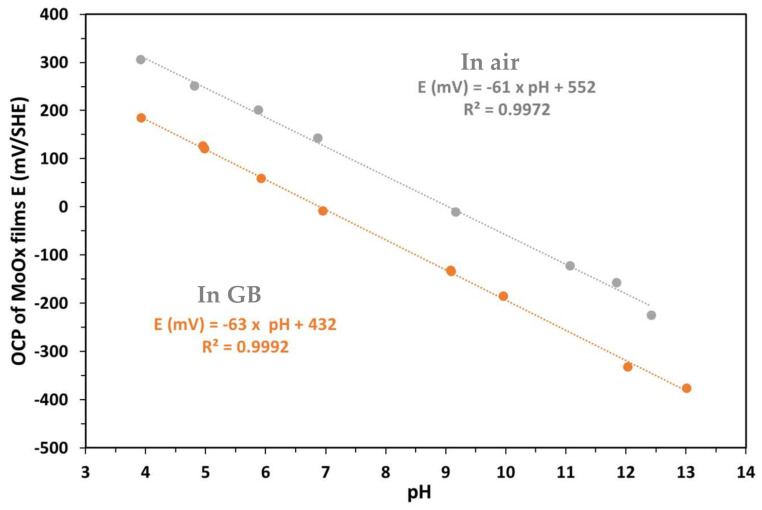
Eh-pH diagram of the MoO_x_ electrode after 70 days of hydration; measurements performed at atmospheric pressure and in GB (pO_2_ = 10^−6^ atm).

Figure 10 provides a comparison between calibration curves obtained in two environments: at atmospheric pressure pO_2_ = 0.2 atm (grey curve) and in a controlled atmosphere in a glove box (pN_2_ = 1 atm and pO_2_ = 10^−6^ atm) (blue curve). The absence of oxygen caused an increase in sensitivity and a decrease in E^0^_Exp_. The increase in electrode sensitivity (from −61 to −63 mV/upH) is due to the absence of O_2_ sorption in the oxygen vacancies within the Mo_2_O_5_ crystalline structure, which provides improved electron conduction at the MoO_x_ film. The presence of these oxygen vacancies, as reported in previous studies on oxides [46,47], improves electron mobility by creating sites for more efficient electron transfer and facilitating redox reactions. This supports more efficient electron transfer within the Mo_2_O_5_ structure, increasing the sensitivity of the electrode, as well as the reproducibility of the measures (Table 5).

The observed decrease in E^0^_Exp_ of about 120 mV in a glove box (GB) may be due to the absence of an oxidation reaction of Mo or MoO_2_. The reason for this is that in the absence of oxygen, there is no further oxidation, which results in a decrease in dissolved species such as HMoO_4_^−^ or MoO_4_^2−^ in the Mo_2_O_5_ coating, causing a decrease in the measured potential. This behavior shows the role of oxygen vacancies in the control of the redox equilibrium of molybdenum species and their impact on the electrochemical properties of the electrode. In addition, the stabilization of the lower oxidation states of molybdenum (Mo (IV) and Mo (V)) in the absence of oxygen limits their conversion to Mo (VI), thus contributing to the reduction in potential.

#### 3.3.4. Influence of Sulfides on the Response of the MoO_x_ Electrode

In order to study the influence of sulfides on our molybdenum electrodes, OCP measurements were carried out using the hydrated electrode in the presence of sulfides (S^2−^ or HS^−^) at different concentrations in a glove box (GB).

**Figure 11:**
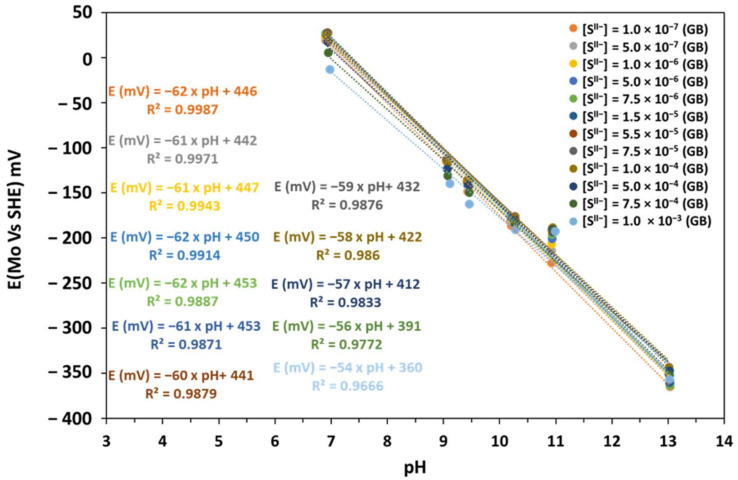
Eh-pH diagram of the MoO_x_ electrode after hydration; measurements performed in a GB and at different sulfide concentrations.

In Figure 11, we observe that the variation in potential ΔE (Mo) is negligible for low sulfide concentrations, which can be observed by the approximation of the calibration curves at these low concentrations. This suggests that interactions of sulfides with the electrode surface are less pronounced in this range. However, at pH = 7, a potential drift of about 37 mV is observed as the sulfide concentration increases from 10^−5^ M to 10^−3^ M, indicating a more significant effect of sulfides at higher concentrations. Although the impact of sulfides remains limited at low concentrations, their influence becomes a little more marked with higher concentrations.

Table 6 presents the statistical treatments of the three experiments on each sulfide concentration on the sensitivity (mV/pH) and the E^0^_Exp_ (mV). The standard deviations for sensitivity (±0.4 to ±0.8 mV/pH) and E^0^_Exp_ (±2.3 to ±10.2 mV) remain within an acceptable range, demonstrating good reproducibility across all sulfide concentrations.

In addition, the sensitivity (slope of the calibration lines) and the E^0^_Exp_ decrease slightly with increasing sulfide concentrations (E (mV) = −62 × pH + 446 for a sulfides concentration of 10^−7^ M to E (mV) = −54 × pH + 360 for a sulfide concentration of 10^−3^ M). This decrease is negligible when the concentration is less than 10^−5^ M (E (mV) = −61 × pH + 453). We suppose that S^2−^ and HS^−^ ions can be adsorbed onto the oxygen vacancies present in the Mo_2_O_5_ structure of our electrode, making complexes with the Mo (VI) or Mo (IV) species on the surface. These complexes slow electron transfer and block active sites.

Li et al. [48] demonstrated that oxygen vacancies and exposed molybdenum (Mo) atoms significantly enhance the adsorption of H_2_S as well as associated sulfide species. They performed density functional theory (DFT)-based calculations and revealed that Mo atoms located at the edges of oxygen-deficient regions exhibit higher adsorption activity due to the reduced shielding effect exerted by external oxygen atoms. This observation [48] is in good agreement with our hypothesis that sulfide ions interact with individual molybdenum centers, forming surface complexes that slow electron transfer and block active sites.

Furthermore, we suggest that the adsorption of sulfide species on the electrode surface results in the formation of a passive film. This layer slows down the electrochemical activity of the electrode and lowers the efficiency of redox reactions occurring on its surface. Erickson et al. [49] reported that under sulfide-rich conditions, molybdate ions (MoO_4_^2−^) undergo a gradual transformation into thiomolybdate species, such as MoS_4_^2−^. These chemical modifications change the surface environment, limiting the number of active sites available for redox reactions. Although thiomolybdate clusters, including [Mo_3_S_13_]^2−^, are well-known for their involvement in hydrogen evolution catalysis (HER) [50], Erickson’s findings emphasize that sulfides can also interact primarily through coordination with individual molybdenum centers. This behavior is especially notable in systems where Mo–Mo bonds are absent [49].

In our case, we suppose that the absence of Mo–Mo bonds and the observed variations in sensitivity and experimental potential E^0^_Exp_ as a function of sulfide concentration mean that the interactions in our system are dominated by sulfide ions. These ions form coordination complexes with individual molybdenum centers. These results are consistent with the proposed mechanism of sulfide adsorption onto oxygen vacancies. Further experiments, such as XPS analysis, could validate this hypothesis.

For reliable pH measurements in the presence of S^−II^, MoO_x_/Mo needs S^−II^ content that can be calculated using calibration curves of OCP values of Ag_2_S/Ag electrodes versus pH at different total concentrations [7,10].

#### 3.3.5. Electrochemical Impedance Spectroscopy Analysis of the MoO_x_/Mo Electrode

**Figure 12:**
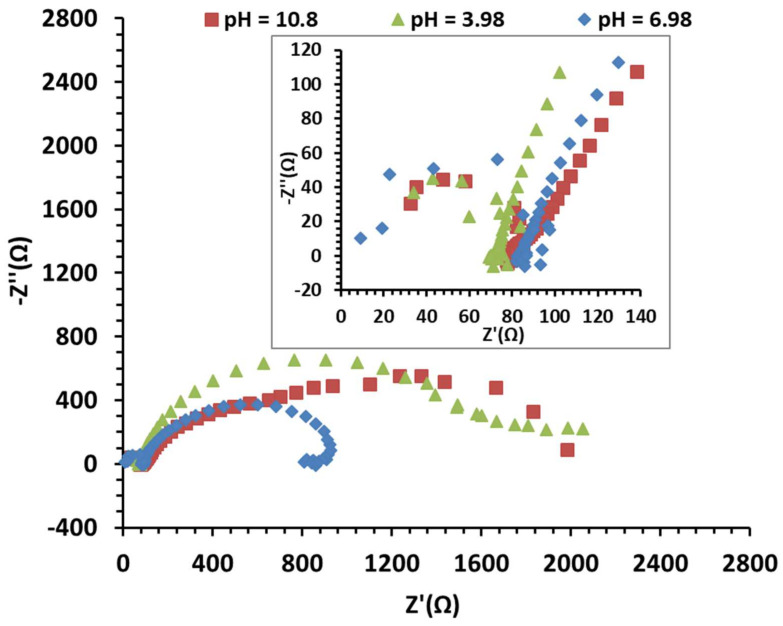
EIS measurements of MoO_x_/Mo film in different buffer solutions, with a focus on high frequencies.

**Figure 13:**
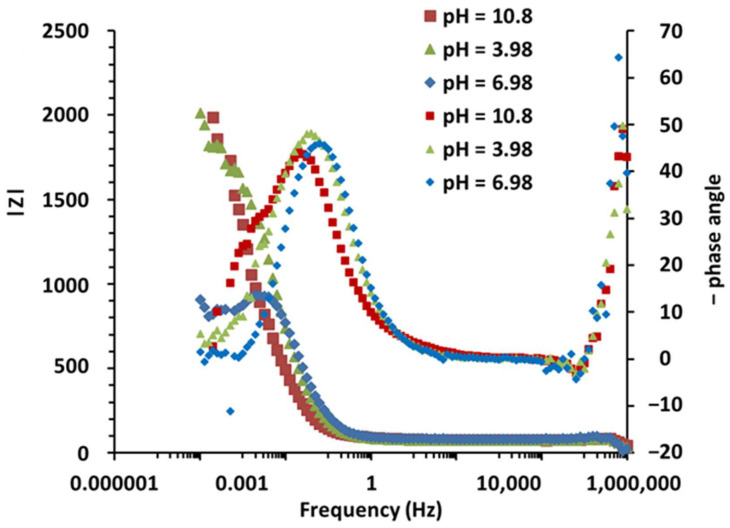
EIS measurements of MoO_x_/Mo film in different buffer solutions. Bode diagram (phase angle) and modulus.

Impedance measurements performed on Mo-MoO_x_-based electrodes in various pH solutions at room temperature shed light on the interface phenomena involved in the response of this electrode to pH variations. The results are presented in the Nyquist and bode diagrams (Figure 12 and Figure 13).

All the curves are characterized by the presence of more than one characteristic time. At first, a small loop appears at very high frequencies (above 50 kHz) under all conditions. This very high-frequency system response can be related to the dielectric dispersion effect of the dielectric material under a rapid electric field [51]. This high frequency capacitance in the electric equivalent circuit will be in parallel with the electrolyte resistance. This response is observed across all conditions. This loop is immediately followed by an inductive effect, still at high frequencies (between 1000 Hz and 50 kHz). These specific adsorption effects at high frequencies may be linked to the presence of multiple interfaces (Mo, MoO_x_ and the electrolyte). Indeed, as current flows, shifts may be created within the oxide layer. Furthermore, the existence of the capacitive effect of the oxide layer can create a transient charge storage effect, which would then create this inductive effect due to rapid rearrangements of the oxide surface in response to H^+^ ions at low pH and OH^−^ at higher pH. For frequencies below 1 kHz, the electrode behavior changes depending on the environment.

At pH = 3.98, between 1 kHz and 1 mHz, a well-defined semi-loop appears at the usual electrochemical double layer frequencies. The resistance associated with the loop is 1778 Ω, and its characteristic frequency is about 3.98 mHz. Some authors have directly linked this loop to the oxide film resistance and the total capacitance of the oxide/electrolyte interface [52]. The loop observed is not perfectly circular, a feature that can be related to the heterogeneity and non-uniformity of the oxide surface [53,54]. We use Constant Phase Element (CPE) instead of the ideal capacitor. The results are fitted using a simple circuit of Randles between 10 mHz and 100 kHz. The capacitance associated with that loop is calculated with

$$C=Y_{0}\;{\left(\omega_{c}\right)}^{n-1}=Y_{0}\;{\left(2\;\pi \;f_{c}\right)}^{n-1}$$

The MoO_x_/Mo pH sensor features a surface area of 0.159 cm^2^. The simulation data summarized in Table 7 indicate a capacitance of *C* = 162.8 mF/cm^2^ at a pH value of 3.98. This capacitance value is remarkably high, comparable to the magnitude observed in super-capacitors [55]. Typically, the capacitance of MoO_x_ is around 150 µF/cm^2^ [34]. This suggests that the observed loop is not only due to the intrinsic capacitance of the MoO_x_ layer but also includes a pseudo-capacitive contribution from redox reactions involving the oxide, the metal and the electrolyte [33,56].

An extra phenomenon appears at lower frequencies (below 1 mHz), indicating an additional pseudo-capacitance induced by reversible redox phenomena such as adsorption or desorption of H^+^ ions and of dissolved species such as ${HMoO}_{4}^{-}$ and ${MoO}_{4}^{2-}$ on the oxide layer, or by the diffusion of the species through the oxide layer.

At pH = 6.98, the characteristic frequency of the main loop is higher (15.8 mHz). The resultant capacitive oxide resistance is therefore about half of the one before (973.5 Ω) and the capacitance of the oxide is 73.9 mF/cm^2^, indicating less charge effects at neutral pH. At the lowest frequencies, a well-defined inductive loop appears, highlighting adsorption phenomena involving species that contribute to increasing the efficiency of the reaction (H^+^ and ${MoO}_{4}^{2-}$). This pseudo-inductive behavior has been reported with a Pt/MoOx/C electrode indicating faradaic processes involving adsorbed intermediates [57,58]. The total polarization resistance of the system, obtained at a frequency of 0.1 mHz, is 800 Ω.

At pH = 10.8, the behavior appears more complex, as a second loop appears at frequencies below 4 mHz, when the main loop associated with the pseudo-capacitance and oxide resistance is not completely defined. This is more visible in the Bode plot representing the impedance phase shift at 1 mHz. The additional process observed at these low frequencies seems to play an important role in the overall system response. The total polarization resistance of the system, due to this supplementary capacitance response, goes from 1751 Ω (oxide resistance) to more than 2000 Ω. The calculated capacitance in this case is 317 mF/cm^2^, with a fitting error of 15%. The electrochemical capacity of molybdenum oxide generally increases with pH, particularly in mildly basic solutions. This is due to protonation/deprotonation reactions.

The hypothesis of an influence of diffusion should thus be considered. Indeed, given the proposed reaction mechanisms and the very low H^+^ concentration at this pH level, the migration of H^+^ ions within the oxide layer that contribute to the electrode’s response can be very slow. A steady state is reached at the lowest frequencies, so a finite diffusion model in the oxide layer or a pseudo-capacitance CPE could be used to model this phenomenon. Otherwise, the new low-frequency interface could also be related to the dissolution phenomenon of MoO_x_ occurring in alkaline environments.

#### 3.3.6. Comparison of MoO_x_/Mo Present Electrode Characteristics with Those of IrO_x_ and RuO_x_ Electrodes in the Literature

Table 8 compares MoO_x_ electrode characteristics of the present work with those of IrOx and RuO_x_ electrodes in the literature [59,60]. This comparison highlights the unique contributions of MoO_x_ electrodes, such as their simple and easily reproducible manufacturing process, their long-term stability in air and water, and their robustness in demanding environments (e.g., in the presence of sulfides) and also in the absence of oxygen. These results highlight the innovation and practical value of MoO_x_-based electrodes for actual applications.

## 4. Conclusions

In this study, the morphology, chemical composition and pH sensing properties of MoO_x_ sensors prepared via thermal oxidation and hydrated in milliQ water were investigated. SEM and EDS results indicate that a MoO_x_ film covers the surface of the molybdenum sensor prepared via thermal oxidation at 500 °C. XPS analysis revealed that the molybdenum substrate is oxidized to Mo (VI). The hydration treatment is crucial to the stability and reliability of the MoO_x_ sensor performance. A 70-day hydration treatment was required to achieve a stable pH response of −61 mV/pH, slightly exceeding the theoretical Nernst value. SEM and EDS analysis showed that the surface is largely covered with MoO_x_, with varying values of “x” at different locations.

XPS analysis after hydration confirmed the presence of Mo (IV) and Mo (VI), indicating a reduction of Mo (VI) to Mo (IV). The performance of the electrodes was studied in the presence and absence of oxygen, and in the absence of oxygen in the presence of sulfides. The absence of oxygen increased sensitivity and reduced the experimental standard potential (E^0^_Exp_), probably due to the effect of oxygen vacancies. The performance of the electrodes in the presence of sulfides showed that the sensors still performed well at low sulfide concentrations. However, at higher concentrations, the formation of complexes with sulfides can reduce electrochemical activity. The thermal oxidation method is therefore recommended for long-term pH monitoring, particularly in environments where oxygen and sulfide concentrations are variable, due to the stability and robustness of MoO_x_ electrodes under such conditions.

## Figures and Tables

**Table 1:** Acid/base buffer pairs and effective pH ranges.

Buffer Species	Effective pH Range
CH_3_COOH/CH_3_COO^−^	4.0–5.6
H_2_PO_4_^−^/HPO_4_^2−^	5.8–7.8
HCO_3_^−^/CO_3_^2−^	8.8–10.8

**Table 2:** Mo and O binding energies in a MoO_x_ film prepared via thermal oxidation.

BE of Mo (3d(5/2), 3d(32))/eV					BE of O1s/eV	
	Mo(VI)	Mo(V)	Mo(IV)s	Mo(IV)u	Mo-O	Absorbed H_2_O/O_2_
Unhydrated	232.88, 236.01	231.60, 234.73	/	/	530.73	532.52
Hydrated	233.10, 236.22	232.54, 235.54	229.53, 232.53	231.23, 234.36	530.64	532.26

**Table 3:** Sensitivity and E^0^_EXP_ at different hydration times of Mo at 500 °C and in air. The experiments were carried out in triplicate, allowing statistical processing to be carried out. For each experiment, the last ten values were used to calculate the mean and the relative standard deviation of OCP and pH; (x = 10; N = 3).

Hydration Time (Days)	Sensitivity (mV/pH)	E^0^_Exp_ (mV)
15	−51.7 ± 0.9	450.7 ± 4.5
45	−58.3 ± 0.5	525.7 ± 3.4
70	−60.3 ± 0.5	541.7 ± 9.0

**Table 4:** OCP of Mo at 500 °C as a function of pH: the values presented are averages between the rise and fall values in pH corresponding to Figure 8 (x = 10; N = 2).

pH	E (mV vs. SHE)
3.910 ± 0.004	296.1 ± 1.5
4.812 ± 0.003	254.1 ± 1.8
5.880 ± 0.004	159.8 ± 3.0
9.173 ± 0.003	−10.6 ± 2.4
11.076 ± 0.004	−123.0 ± 1.1
12.425 ± 0.003	−222.4 ± 4.2

**Table 5:** Sensitivity and E^0^_Exp_ at 70 days of hydration of Mo at 500 °C in air and in a glove box (x = 10; N = 3).

70 Days of Hydration	Sensitivity (mV/pH)	E^0^_Exp_
In Air	−60.3 ± 0.5	541.7 ± 9.0
In GB	−62.3 ± 0.9	435.3 ± 4.7

**Table 6:** Sensitivity and E^0^_Exp_ at 70 days of hydration of Mo at 500 °C in a glove box and in the presence of different concentrations of sulfide (from 10^−7^ to 10^−3^ M). The experiments were carried out in triplicate.

Concentration of Sulfides (M)	Sensitivity (mV/pH)	E^0^_Exp_ (mV)
1.0 × 10^−7^	−62.5 ± 0.4	449.2 ± 2.3
5.0 × 10^−7^	−61.6 ± 0.5	445.8 ± 3.2
1.0 × 10^6^	−61.9 ± 0.8	452.1 ± 4.8
5.0 × 10^−6^	−62.3 ± 0.6	455.9 ± 6.0
7.5 × 10^−6^	−62.5 ± 0.8	460.3 ± 7.4
1.0 × 10^−5^	−62.4 ± 0.8	460.6 ± 8.0
5.0 × 10^−5^	−61.2 ± 0.7	449.2 ± 8.4
7.5 × 10^−5^	−60.2 ± 0.7	440.1 ± 8.2
1.0 × 10^−4^	−59.2 ± 0.7	425.2 ± 2.3
5.0 × 10^−4^	−58.5 ± 0.7	410.7 ± 5.9
7.5 × 10^−4^	−57.1 ± 0.7	386.8 ± 10.2
1.0 × 10^−3^	−55.4 ± 0.8	366.8 ± 5.2

**Table 7:** Simulation results for Randles circuit fitting of MoO_x_/Mo pH sensors (10 mHz to 100 kHz).

pH	pH = 3.98	Error (%)	pH = 6.98	Error (%)	pH = 10.8	Error (%)
Rs	72.11	0.302	84.34	0.329	78.13	0.505
Q-Yo	0.01602	0.964	0.007963	0.885	0.01422	1.5222
Q-n	0.8698	0.658	0.8312	0.695	0.6948	1.556
Rox (Ω)	1778	5.718	973.5	2.393	1751	15.53
Fc (Hz)	0.0039817	/	0.01584893	/	0.00251189	/
Capacitance (F)	0.02589521	/	0.01175395	/	0.05044465	/
Capacitance (mF/cm^2^)	162.8	/	73.90	/	317.18	/

**Table 8:** Comparison of the MoOx electrode characteristics from this study with those of IrOx and RuOx electrodes reported in the literature [59,60].

Property	MoO_x_ (in This Work)	IrO_x_ [59]	RuO_x_ [60]	Unique Advantages of MoO_x_
Type of electrode fabrication	Heat treatment	Thermal cycling	Screen printing + drying (120 °C) + firing (850 °C, 30 min)	Simple, cost-effective
Storage in water	Yes (70 days)	Initial storage in water, periodic calibration over 200 days	Water: Stable, Air: Unstable	Long-term stability
Sensitivity (mV/pH)	~−61	−64.6	−60.78 mV/pH (water storage), −56.38 mV/pH (air storage)	Comparable to RuO_x_ and IrO_x_
Stability	stable	stable	Water: Stable, Air: Unstable	Stable
pH range	4–13	1.68–10.01	2–10	Demonstrated robustness across a wide pH range
Glove box study (GB)	conducted (Section 3.3.3)	not reported	not reported	Tested in controlled environments
GB with varying [S^2−^] levels	conducted (Section 3.3.4)	not reported	not reported	Resistant to sulfide interference
Cost	low	high	high	Low-cost materials and simple preparation

## Data Availability

The data presented in this study are available upon request from the corresponding authors.
